# Supplementary figures and images for: Characterization of cerebrospinal fluid (CSF) microbiota from patients with CSF shunt infection and reinfection using high throughput sequencing of 16S ribosomal RNAgenes
Source: PLoS One. 2021 Jan 6;16(1):e0244643. doi: 10.1371/journal.pone.0244643 (PMC7787469; doi:10.1371/journal.pone.0244643)

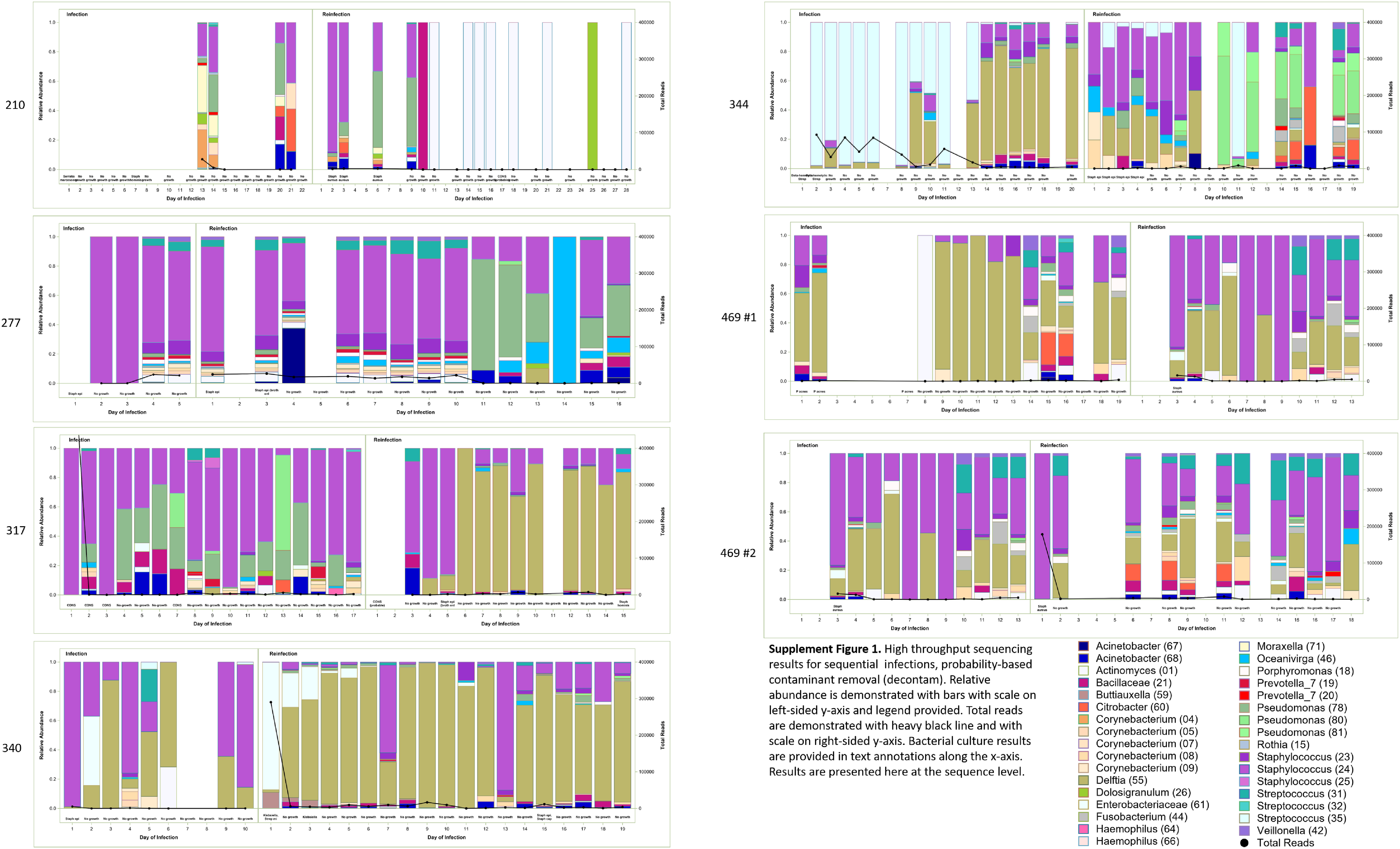

Supplement: S1 Fig — Relative abundance is demonstrated with bars with scale on left-sided y-axis and legend provided. Total reads are demonstrated with heavy black line and with scale on right-sided y-axis. Bacterial culture results are provided in text annotations along the x-axis. While the analysis was conducted at sequence level, results are presented here at the genus level for ease of interpretability. (TIF) [file pone.0244643.s001.tif]

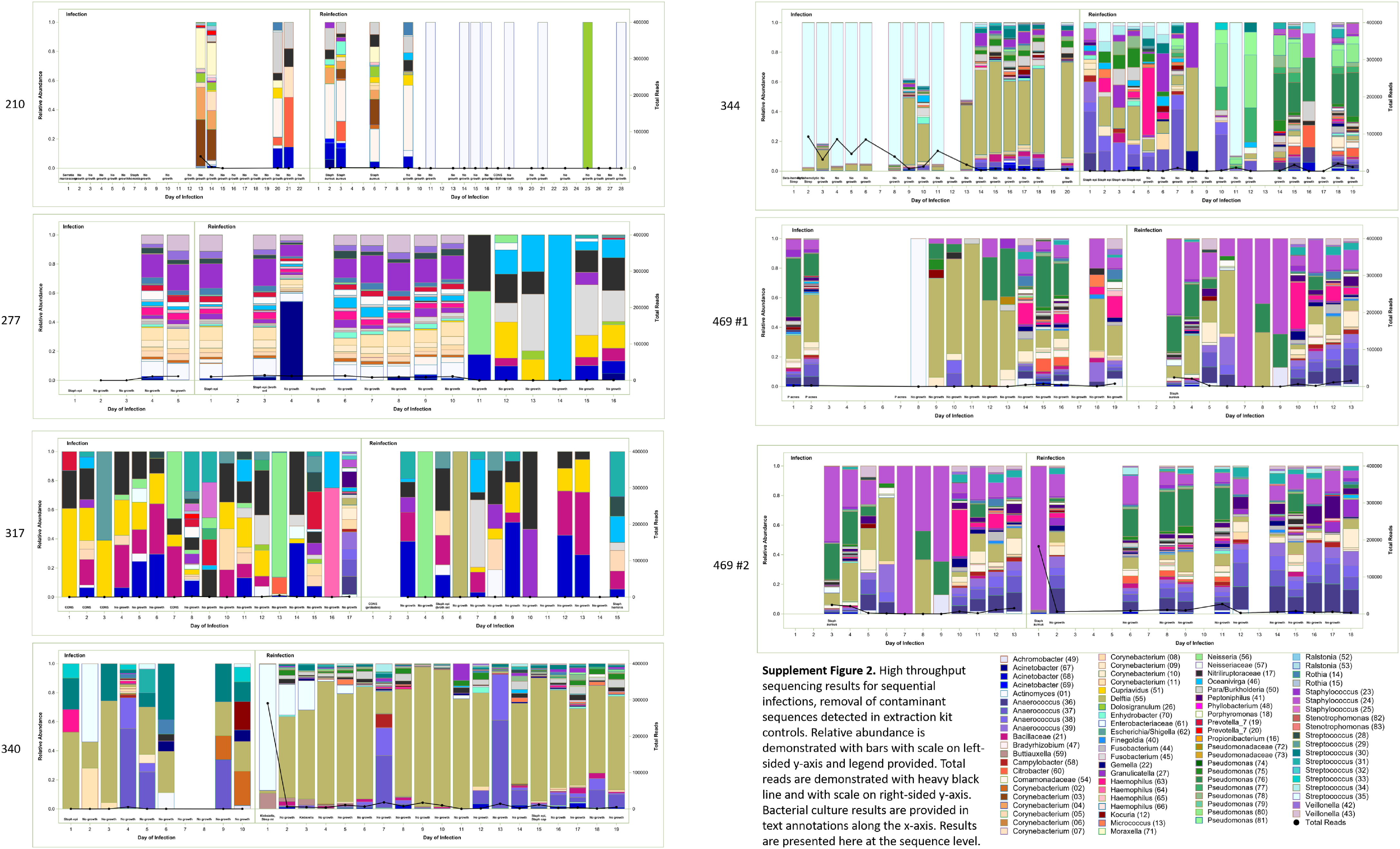

Supplement: S2 Fig — Relative abundance is demonstrated with bars with scale on left-sided y-axis and legend provided. Total reads are demonstrated with heavy black line and with scale on right-sided y-axis. Bacterial culture results are provided in text annotations along the x-axis. Results are presented here at the sequence level. (TIF) [file pone.0244643.s002.tif]
