# Supplementary material for: Characterization of cerebrospinal fluid (CSF) microbiota from patients with CSF shunt infection and reinfection using high throughput sequencing of 16S ribosomal RNAgenes
Source: PLoS One. 2021 Jan 6;16(1):e0244643. doi: 10.1371/journal.pone.0244643 (PMC7787469; doi:10.1371/journal.pone.0244643)
Supplement: Attachment — Submitted filename: Response to Reviewers.docx [file pone.0244643.s003.docx]

October 26, 2020

Dear PLOS One Editorial team,

We appreciate the consideration given by your reviewers and the editorial board to our original research article PONE-D-19-35359 entitled “Characterization of cerebrospinal fluid (CSF) microbiota from patients with CSF shunt infection and reinfection using high throughput sequencing of 16S ribosomal RNA genes”.

We are responding to the critiques provided with the following changes (original feedback is provided in italics):

1. *Journal requirements: Please ensure that your manuscript meets PLOS ONE's style requirements, including those for file naming.*

Figure files and supporting information were reformatted and renamed to comply with guidelines. In addition, edits were made to the title page to comply with PLOS One formatting requirements.

2. *Journal requirements: In the ethics statement in the Methods and online submission information, please ensure that you have specified what type of informed consent you obtained (for instance, written or verbal, and if verbal, how it was documented and witnessed). Given that your study included minors, state whether you obtained consent from parents or guardians.*

We have added clarification that “written consent was obtained from parents or guardians, and assent when age- and developmentally-appropriate from study subjects” to the ethics statement.

3. *Journal requirements: We note that you are reporting an analysis of a microarray, next-generation sequencing, or deep sequencing data set. PLOS requires that authors comply with field-specific standards for preparation, recording, and deposition of data in repositories appropriate to their field.*

We were able to upload our data to NCBI Sequence Read Archive PRJNA608523 on February 24, 2020 with a release date in June 2020.

4. *Journal requirements: We note that you have stated that you will provide repository information for your data at acceptance. Should your manuscript be accepted for publication, we will hold it until you provide the relevant accession numbers or DOIs necessary to access your data. If you wish to make changes to your Data Availability statement, please describe these changes in your cover letter and we will update your Data Availability statement to reflect the information you provide.*

We were able to upload our data to NCBI Sequence Read Archive PRJNA608523 on February 24, 2020 with a release date in June 2020. We have revised the cover letter accordingly.

*5. Journal requirements: Thank you for stating the following in the Financial Disclosure section:*

*'This study was supported by the National Institutes of Health via R01 NS095979 awarded to TDS, CEP, PH, LRH, DLL, PM. DLL receives research funding and equipment for unrelated projects through Microbot Medical, Inc. and Medtronic, Inc. LRH receives additional support from the National Institutes of Health via K24 HL141669.  The funders had no role in study design, data collection and analysis, decision to publish, or preparation of the manuscript.' We note that you received funding from a commercial source: Microbot Medical, Inc. and Medtronic, Inc*

*a. Please provide an amended Competing Interests Statement that explicitly states this commercial funder, along with any other* [*relevant declarations*](about:blank) *relating to employment, consultancy, patents, products in development, marketed products, etc.*

*Within this Competing Interests Statement, please confirm that this does not alter your adherence to all PLOS ONE policies on sharing data and materials by including the following statement: "This does not alter our adherence to PLOS ONE policies on sharing data and materials.”*

We have included the two commercial sources above in Dr. Limbrick’s funding disclosures on the PLOSOne website. We have provided the financial disclosure statement above in the cover letter, and provided the additional statement according to the above guidance.

*b. Please include your amended Competing Interests Statement within your cover letter. We will change the online submission form on your behalf.*

 We have included the two commercial sources above in Dr. Limbrick’s funding disclosures on the PLOSOne website. We have provided the financial disclosure statement above in the cover letter, and provided the additional statement according to the above guidance. We appreciate the additional support.

*6. We note you have included a table to which you do not refer in the text of your manuscript. Please ensure that you refer to Table 1 in your text; if accepted, production will need this reference to link the reader to the Table.*

 	We have revised an incorrect reference to Table S1 to a correct reference to Table 1.

*7. Please upload a copy of Supporting Information Table S1 which you refer to in your text on page 9.*

 	We have revised an incorrect reference to Table S1 to a correct reference to Table 1.

*8. Reviewer #1, general comments: The authors have used proper controls were possible (extraction kit control, PCR and sequencing control). However, I have main concerns regarding the existence of contaminant sequences in the dataset even after authors’ attempt to remove these contaminants using a popular bioinformatics package (decontam). CSF is considered an sterile biological fluid, and even in case of infection, authors should have expected very low bacterial load in samples. Reagent contaminations could have been minimized prior to extraction and sequencing. A more stringent removal of contaminant reads is required to properly interpret the results.*

We appreciate this feedback and have provided additional analyses using the more stringent approach suggested. We have revised the methods section accordingly, provided new results in a new Figure 4 and the original Table 4. We did observe a statistically significant association using this approach, and hence have revised the results and discussion accordingly. Please see specific comments below for additional details about revisions made.

*9. Reviewer #1, general comments: Also, bioinformatics analyses should focus on taking advantage of the information of sequence variants rather than using bacterial genus level.*

We appreciate this feedback. The original analysis was conducted at the sequence level, and original figures presented at genus level for interpretability. We have now provided results at the sequence level in the Supplemental Figures. We have also provided this clarification in revisions to the methods section as well as figure legends. Please see specific comments below for additional details about revisions made.

*10. Reviewer #1, specific comments: Abstract: Lines 33-34: not clear how many samples from each patient? 2 in total, or at the end of each (re)infection and begging of next reinfection? This needs to be clarified in the methods section as well.*

We have revised the abstract methods to read, “Serial CSF samples were obtained from 6 patients, 5 with 2 infections and 1 with 3 infections; the study was limited to those for which CSF samples were available from the end of infection and beginning of reinfection.”

We included the following statement in the Study Subjects section of the Methods as well: “We analyzed serial CSF samples from 6 patients, 5 with 2 infections and 1 with 3 infections; the study was limited to those for which CSF samples were available from the end of infection and beginning of reinfection.”

*11. Reviewer #1, specific comments: Abstract Lines 38-39: need to briefly clarify the method by which 8 taxa were detected? HTS or culture? Are these unique amplicon sequence variants (ASVs)?*
We have now clarified that 8 taxa were identified using HTS. These were analyzed at a sequence level; figures were originally presented at genus level for interpretability. The original analysis was conducted at the sequence level, and original figures presented at genus level for interpretability. We have now provided results at the sequence level in the Supplemental Figures.

*12. Reviewer #1, specific comments: Introduction: Line 71: “over 2,000 CSF shunt infections diagnosed each year” worldwide or the US?*
We have now clarified that there are over 2,000 shunt infections annually in the U.S.

*13. Reviewer #1, specific comments: In the introduction, please provide information regarding pathogens/opportunistic commonly isolated from CSF shunt infections (if any literature available).*

Thank you. We have added a statement: “The most common pathogens recovered from conventional microbiologic cultures include primarily *Staphylococcus epidermidis* and *Staphylococcus aureus*, followed by gram-negative organisms.[ref]”

*14. Reviewer #1, specific comments: Methods: Lines 115-123: Still not clear how many samples were collected per patient? In line 105, authors indicate “we examined serial CSF obtained from the subset of children”, but never refer to exact numbers until lines 243-245. Please clarify this in the “specimen collection” section.*

We included the following statement: “We analyzed serial CSF samples from 6 patients, 5 with 2 infections and 1 with 3 infections; the study was limited to those for which CSF samples were available from the end of infection and beginning of reinfection.” We elected to update the Study Subjects section rather than the Specimen Collection section of the Methods.

*15. Reviewer #1, specific comments: Lines 129-134: would be informative to provide a one line description of the conventional culture (media used, incubation time and temperature).*

Because the media and incubation approaches can differ based on organism recovered, we have elected to retain the original description: “Conventional cultures are the traditional diagnostic approach used to detect typical pathogens in infectious diseases and were performed in a clinical microbiology laboratory following Clinical and Laboratory Standards Institute guidelines”. We had included in the limitation section the following statement as well: “Testing from the hospital-certified laboratories was limited to routine aerobic cultures, practices that are subject to individual laboratory practices, including policies affecting identification of all organisms on a plate, incubation for extended periods of time, and enrichment for slow growing and fastidious organisms. We did not have information about variability or density of colonies on the original plates.”

*16. Reviewer #1, specific comments: Results: Lines 272-273: Color codes used in Figure1 are a bit difficult to differentiate, but apparently even after removal of the kit contaminant sequences, reads belonging to important taxa such as Staphylococcus, Streptococcus, Pseudomonas, and Corynebacterium remain in the dataset. Based on Figure 3, these bacterial taxa are abundant in the final microbiota profile of biological samples. There are main limitations here:
- Taxa indicated in the bar charts, are these based on the proportion of bacterial genera (genus level) or ASV? If based on genera, the results are simply not acceptable. You are losing resolution, you could have streptococcal ASVs in the kit contaminant with different sequence than those detected in biological samples. Combing information at genus level is misleading. IF the information provided is based on genus level, please reanalyze the data and present it at the ASV level (both with respect to bar charts and PCoA analysis).*

We appreciate that we need to be far more explicit about our approach. We have added the following statement to the Data Analysis section: “While the analysis was conducted at sequence level, Figures 1, 3, and 4 present results at the genus level for ease of interpretability.”

We have also added results at the sequence level and go on to say, “Supplemental Figures 1 and 2 present the same results at the sequence level.”

We have edited the legends to Figures 1, 3, and 4.

We have also revised the limitation section of the discussion to read, “Decontam R effectively removed some kitome sequences, and similar results were observed following removal of contaminant sequences detected in extraction kit controls.”

*17. Reviewer #1, specific comments: Results: - The decontam package was not developed to (and is not able to) completely fix the issue of laboratory reagent contamination, particularly when it comes to low biomass samples. Most of the taxa detected in reagents are successful environmental survivors capable of producing biofilms. Members of these taxa are also omnipresent in hospitals, can be potential pathogens causing human diseases, or even found as opportunistic inside or on different surfaces of our body (e.g. skin). With respect to CFS, which is considered an sterile biological fluid, one should expect zero to very low levels of bacterial DNA even in the case of CSF shunt infections. The proper way to address this issue was to pre-treat extraction kit and PCR reagents with DNAse prior to conducting the experiment (there are even commercial kits available for this purpose). Now that this method has been neglected, the most proper way to analyze* *a sensitive dataset like CSF is to completely remove contaminant ASVs detected in extraction kit controls from the biological samples. Then report the total number of reads and microbiota profile before and after removal of contaminant ASVs (this can be done and presented in parallel to the of decontam to enable unbiased comparison). Of course, this approach could lead to removal of biologically relevant ASVs originating from samples, but this is the only way to make the results reliable.*

Thank you for the suggestion. We have now provided additional analyses using the more stringent approach suggested. We have revised the methods section to explain, “Two strategies were used to address contaminant sequences. … Alternately, sequences detected in extraction kit controls were identified as contaminants and were removed from the CSF samples.”

We have provided results in a new Figure 4. The results section now reads, “Figure 3 presents results following probability-based contaminant removal (decontam). Similar results were observed following removal of contaminant sequences detected in extraction kit controls (Figure 4).”

We have also updated the Chao-Jaccard abundance-based similarity index in Table 4. The associated text in the Results section now reads, “Following removal of contaminant sequences observed in extraction kit controls, median Chao-Jaccard abundance-based similarity index for matched infection pairs was 0.33 (IQR 0.25-0.53) compared to that of unmatched pairs of 0.17 (IQR 0.12-0.32) [p = 0.04].”

We did observe a statistically significant association using this approach, and hence have revised the results section accordingly: “Therefore, CSF microbiota at the end of infection and beginning of reinfection were more correlated than in any two unrelated infections after removal of all sequences observed in negative control samples. We reject the hypothesis that sequential infections were independent.”

The discussion and conclusion have been revised to state that the high throughput sequencing results also suggest that microbiota of consecutive infection episodes may be associated.

*18. Reviewer #1, specific comments: Results: - A concerning point in the post-decontam microbiota profile of biological samples is the presence of several bacterial groups that should have been detected by aerobic culture (most members of staphylococcus, pseudomonas, coryebacterium, etc. are not fastidious and grow aerobically). The absence of these taxa from culture results is additional proof for possibility of contamination.*
We appreciate this feedback and have provided additional analyses using the more stringent approach suggested. As already described, we have revised the methods section accordingly, provided new results in a new Figure 4 and the original Table 4. We did observe a statistically significant association using this approach, and hence have revised the results and discussion accordingly.

We also would suggest that high throughput sequencing detects bacterial DNA rather than culturable cells. We had previously stated in the discussion section, “Detection of microbial DNA, which could be from either viable or nonviable cells, by PCR does not guarantee that living bacteria or fungi were present at the time of sample collection.” We have now added the statement, “Alternately, high throughput sequencing may be detecting small colony variants and/or bacteria whose growth is being suppressed by antibiotics.”

*19. Reviewer #1, specific comments: Results: - Next limitation of decontam package or even complete removal of contaminant DNAs is between-kit variation in the profile of contaminant DNA. In Figure1A (and less in Figure1B) it is evident that each batch of kit has it’s own microbiota profile, making removal of contaminant reads more challenging. So, when re-analyzing the dataset by complete removal of contaminant DNA, each biological sample should be filtered based on its corresponding extraction kit profile.*

We appreciate this feedback and have provided additional analyses exactly as suggested.

*20. Reviewer #1, specific comments: Results: Lines 287-289: “indicating decontam removed contaminating signal that originated from the DNA extraction kits”. Not necessarily, significant clustering pattern does not equal to removal of contaminant DNA. As indicated, you have shared contaminant taxa (and most likely ASVs) in your extraction kit control and biological samples even after using decontam.*

We appreciate this feedback and have provided additional analyses using the more stringent approach suggested. As already described, we have revised the methods section accordingly, provided new results in a new Figure 4 and the original Table 4. We did observe a statistically significant association using this approach, and hence have revised the results and discussion accordingly.

*21. Reviewer #1, specific comments: Results: Lines 294-297: Why doing beta-diversity analysis at the genus level? You have used dada2 algorithm and have access to ASVs, please use that data.*

We appreciate this feedback. The original analysis was conducted at the sequence level, and original figures presented at genus level for interpretability. We have now provided results at the sequence level in the Supplemental Figures. We have also provided this clarification in revisions to the methods section as well as figure legends. Please see specific comments above for additional details about revisions made.

*22. Reviewer #1, specific comments: Results: Figure2: Also indicate on the PCoA graph the kit control and negative controls after the decontam package.*

We appreciate this suggestion, and affirm that Figure 2 provides the reader feedback on the effectiveness of decontam for contaminant removal. Because we have performed the additional analysis with complete removal of contaminant sequences, we believe this suggestion is less critical and have elected to defer.

*23. Reviewer #1, specific comments: Results: Line 301-303: Again, please provide information whether the streptococcal, staylococcal, pseudomonas, and Corynebacterial ASVs remaining in the kit extraction control after decontam are the same/different from the ones detected in post-decontam microbiota profile of the biological samples.*

Thank you for this question. While decontam removed many sequences that were apparent in extraction kit control samples, some of the sequences present in extraction kit controls were also detected in the post-decontam microbiota profile of the biological samples. Of 10 Corynebacterial ASVs, 5 were removed via decontam. Of the remaining sequences, 4 were not present in any extraction kit control sample; a single sequence was present in extraction kit 2 only. Of 8 Streptococcal ASVs, 5 were removed via decontam. Of the remaining sequences, 2 were not present in any extraction kit control sample; a single sequence was present in extraction kit 1 only. Of 3 Staphylococcal ASVs, none was removed by decontam. One of the sequences was not present in any extraction kit controls; 1 was present in kit 2 only; and 1 was present in kits 1, 2, and 3. Of 8 Pseudomonas ASVs, 4 were removed via decontam. Of the remaining sequences, 3 did not appears in any extraction kit control sample; a single sequence was present in extraction kits 1 and 2.

*24. Reviewer #1, specific comments: Results: Lines 311-314: Regardless of whether performed on the same flowcell or not, sequencing reads cannot be used for quantification. There are many technological limitations to this, please revise this section.*

We appreciate the reviewers point and have removed this statement.

*25. Reviewer #1, specific comments: Discussion: The authors have identified and acknowledged potential limitations of the study. However, as indicated earlier, most of the results/discussion of this manuscript is based on the ability of decontam package to completely remove contaminant reads, which is not a true statement. Particularly, the authors need to expand this limitation by discussing why standard culture failed to recover supposedly aerobic taxa detected by sequencing.*
The reviewer raises an excellent point. We have revised the limitations to reflect the additional analysis, and it now reads, “Decontam R effectively removed some kitome sequences, and similar results were observed following removal of contaminant sequences detected in extraction kit controls.”

We also would suggest that high throughput sequencing detects bacterial DNA rather than culturable cells. We had previously stated in the discussion section, “Detection of microbial DNA, which could be from either viable or nonviable cells, by PCR does not guarantee that living bacteria or fungi were present at the time of sample collection.” We have now added the statement, “Alternately, high throughput sequencing may be detecting small colony variants and/or bacteria whose growth is being suppressed by antibiotics.”

*26. Reviewer #1, specific comments: Data availability: The sequencing reads and metadata (sample information) should become available to allow careful examination of the results.*

We were able to upload our data to NCBI Sequence Read Archive PRJNA608523 on February 24, 2020 with a release date in June 2020.

*27. Reviewer #2: 1. It is not clear why only the V4 region of 16S rRNA gene was sequenced and why metagenomic sequencing was not performed.*

We chose the 16S region because it covers bacterial taxa cultured from CSF and there are a substantial number of human sequences present. We are exploring metagenomics as a new direction, and have added the following statement to the discussion: “In addition, future work will need to explore alternate sequencing approaches, including metagenomic sequencing.”.

*28. Reviewer #2: 2. It is very difficult to assess the importance of the relative abundance of the microbial contents of the sample for which the total microbial amount is uncertain. The microbial burden of each sample should be more reliably and accurately determined. Sequencing reads are inappropriate for quantification purposes. And, relative abundance does not speak to total abundance.*

We acknowledge the limitations of total reads, and hence present them beside standard quantification using quantitative 16S PCR results in Table 3. As shown, quantitative PCR is rarely detected in CSF shunt infection, so total reads are presented to provide the reader additional indirect evidence to the low bacterial load present. We have also removed the statement in reference to figure 3 that read, “Because all the samples were analyzed on the same flow cell, total reads are able to be compared.”

*29. Reviewer #2: 3. Use of the Decontam program does not convincingly remove all contaminating sequences such that a good quality representation of the actual sample is obtained. It is surprising that sequences for aerobic, relatively hardy taxa were found in the 16S dataset but not detected in the cultured dataset. This strongly suggests the possibility of the results coming from contamination. This concern is further supported by the results shown in Figure 1, where each kit batch has a different profile.*

Thank you for the suggestion. We have now provided additional analyses using a more stringent approach. We have revised the methods section to explain, “Two strategies were used to address contaminant sequences. … Alternately, sequences detected in extraction kit controls were identified as contaminants and were removed from the CSF samples.”

We have provided results in a new Figure 4. The results section now reads, “Figure 3 presents results following probability-based contaminant removal (decontam). Similar results were observed following removal of contaminant sequences detected in extraction kit controls (Figure 4).”

We have also updated the Chao-Jaccard abundance-based similarity index in Table 4. The associated text in the Results section now reads, “Following removal of contaminant sequences observed in extraction kit controls, median Chao-Jaccard abundance-based similarity index for matched infection pairs was 0.33 (IQR 0.25-0.53) compared to that of unmatched pairs of 0.17 (IQR 0.12-0.32) [p = 0.04].”

We did observe a statistically significant association using this approach, and hence have revised the results section accordingly: “Therefore, CSF microbiota at the end of infection and beginning of reinfection were more correlated than in any two unrelated infections after removal of all sequences observed in negative control samples. We reject the hypothesis that sequential infections were independent.”

The discussion and conclusion have been revised to state that the high throughput sequencing results also suggest that microbiota of consecutive infection episodes may be associated.

*30. Reviewer #2: 4. It appears that the figures are showing results of taxa based on genus level not ASV. Why was this done? ASV is higher resolution and should be able to distinguish contaminant reads from sample reads.*

We appreciate that we need to be far more explicit about our approach. We have added the following statement to the Data Analysis section: “While the analysis was conducted at sequence level, Figures 1, 3, and 4 present results at the genus level for ease of interpretability.”

We have also added results at the sequence level and go on to say, “Supplemental Figures 1 and 2 present the same results at the sequence level.”

We have similarly edited the legends to Figures 1, 3, and 4.

*31. Reviewer #2: 5. The description of the number of samples collected and analyzed per patient and which ones exactly were used for analysis is not clear. After much thought it is possible to figure it out, but more clarity would be helpful.*

We have revised the abstract methods to read, “Serial CSF samples were obtained from 6 patients, 5 with 2 infections and 1 with 3 infections; the study was limited to those for which CSF samples were available from the end of infection and beginning of reinfection.”

We included the following statement in the Study Subjects section of the Methods as well: “We analyzed serial CSF samples from 6 patients, 5 with 2 infections and 1 with 3 infections; the study was limited to those for which CSF samples were available from the end of infection and beginning of reinfection.”

*32. Reviewer #2: 6. For the discussion, it would be helpful to provide more comparative information of the results from the current study with the types of microbes frequently found associated with CSF infections/reinfections, based on the literature. Since there are over 2000 cases every year (assuming in US??), one would think that there would be information regarding the types of microbes associated with these infections. Are there aerobic bacteria often found?*

Thank you. We have added a statement: “The most common pathogens recovered from conventional microbiologic cultures include primarily *Staphylococcus epidermidis* and *Staphylococcus aureus*, followed by gram-negative organisms.[ref]”

We appreciate the suggestions and look forward to your response.

Tamara Simon
